# Sacral Ewing Sarcoma Presenting With Cauda Equina Syndrome: A Case Report and Radiological Review

**DOI:** 10.7759/cureus.106794

**Published:** 2026-04-10

**Authors:** Amrit Pun, Yung Kong Wong, Pui Lam Cheung, Wing Ho Chong, On Chee Li

**Affiliations:** 1 Department of Radiology, Tuen Mun Hospital, Tuen Mun, HKG

**Keywords:** cauda equina syndrome (ces), ewing sarcoma (es), neurogenic bladder dysfunction, pediatric ewing sarcoma, sacral ewing sarcoma

## Abstract

Ewing sarcoma is an aggressive neoplasm that primarily affects children and young adults. Due to the complex anatomy of the sacral region and the proximity to critical neurovascular structures, these tumors often present a significant diagnostic and therapeutic challenge. In this case, the patient initially presented with lower back pain before worsening and presenting with cauda equina syndrome. This is a rather rare case of sacral Ewing sarcoma presenting with cauda equina syndrome in a pediatric patient. A high index of clinical suspicion and timely imaging play a vital role in diagnosis and eventually prognosis.

## Introduction

After osteosarcoma, Ewing sarcoma is the second most frequent primary bone malignancy in children, typically affecting the shafts of long bones or flat bones, such as the ilium, scapula, and chest wall [1]. Survival rates for this tumor, which was once universally lethal, have now improved, though they still differ depending on the tumor's location [1]. Long-term survival rates reach as high as 86% for spinal tumors, whereas they are only 25% for sacrococcygeal tumors [1,2]. A retrospective review of the National Cancer Database in September 2022 for all pediatric Ewing sarcoma cases revealed an overall five-year survival rate of 74.5%, with rates of 84.7% for localized disease and 50.4% for those presenting with metastatic disease [3]. Since many patients who appear to have only localized disease at diagnosis may actually harbor hidden (systemic) metastases, all patients require both multidrug chemotherapy and local control measures, such as surgery or radiation therapy [4]. Prompt diagnosis and timely initiation of treatment are essential for achieving a favorable prognosis. Cauda equina syndrome, a rare condition caused by compression of the cauda equina nerves, typically presents with lower back pain, sciatica, motor or sensory deficits, and impaired visceral function - most notably loss of bladder and bowel control. Ewing sarcoma is an uncommon cause of cauda equina syndrome.

This case documents a pediatric patient presenting with cauda equina syndrome; imaging showed a sacral lesion, which was later proven to be Ewing sarcoma. This report discusses radiological assessment of the tumor, cauda equina syndrome, and post treatment changes using several imaging modalities.

## Case presentation

A nine-year-old Asian male initially presented with a two-month history of lower back pain. He subsequently developed acute urinary retention, constipation, reduced anal sphincter tone, and lower extremity weakness graded 1-2 out of 5 on the Medical Research Council Scale for Muscle Strength [5].

**Figure 1:**
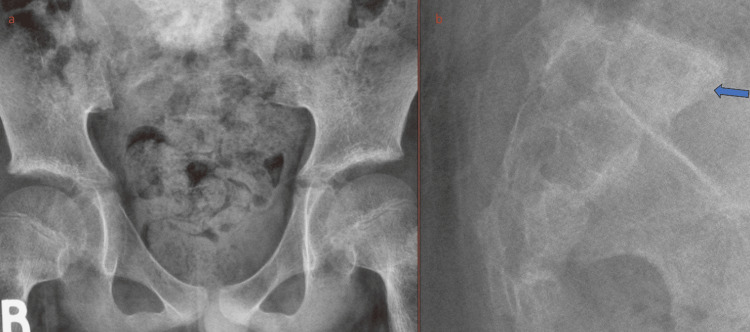
Frontal (a) and lateral (b) radiographs of the sacrum showing vague increased sclerosis of the S1 vertebra (blue arrow).

**Figure 2:**
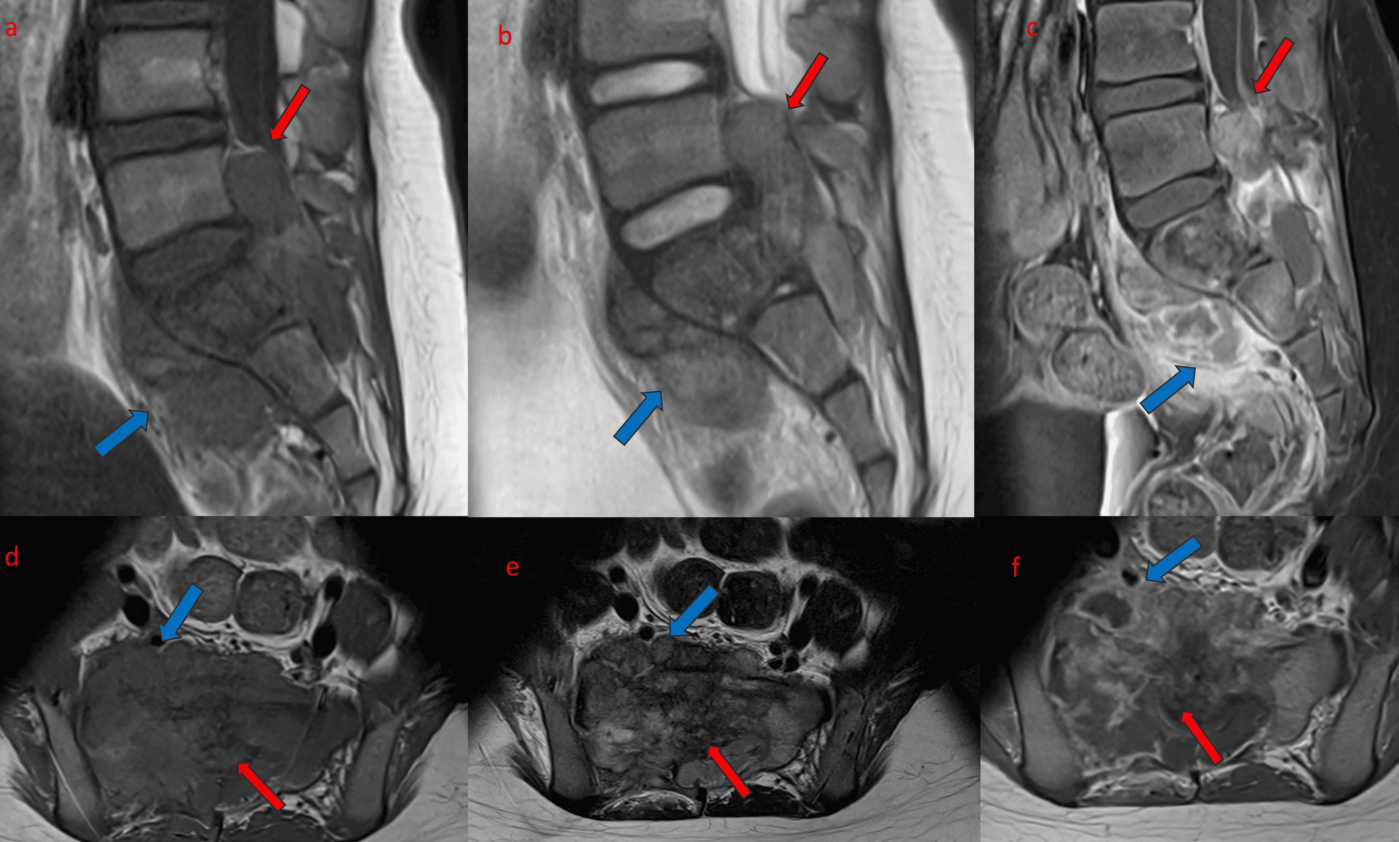
Selected sagittal (a-c) and axial (d-f) MRI images (left to right: T1W, T2W, and T1W post contrast). Heterogeneously enhancing, T1 hypointense, T2 heterogeneous signal intensity irregular mass arising from the S1 vertebra, with extraosseous soft tissue extension into the pelvis (blue arrow) and spinal canal (red arrow).

Initial screening radiograph showed vague increased sclerosis of the S1 vertebra, more conspicuous on the lateral view (Figure 2).

**Figure 3:**
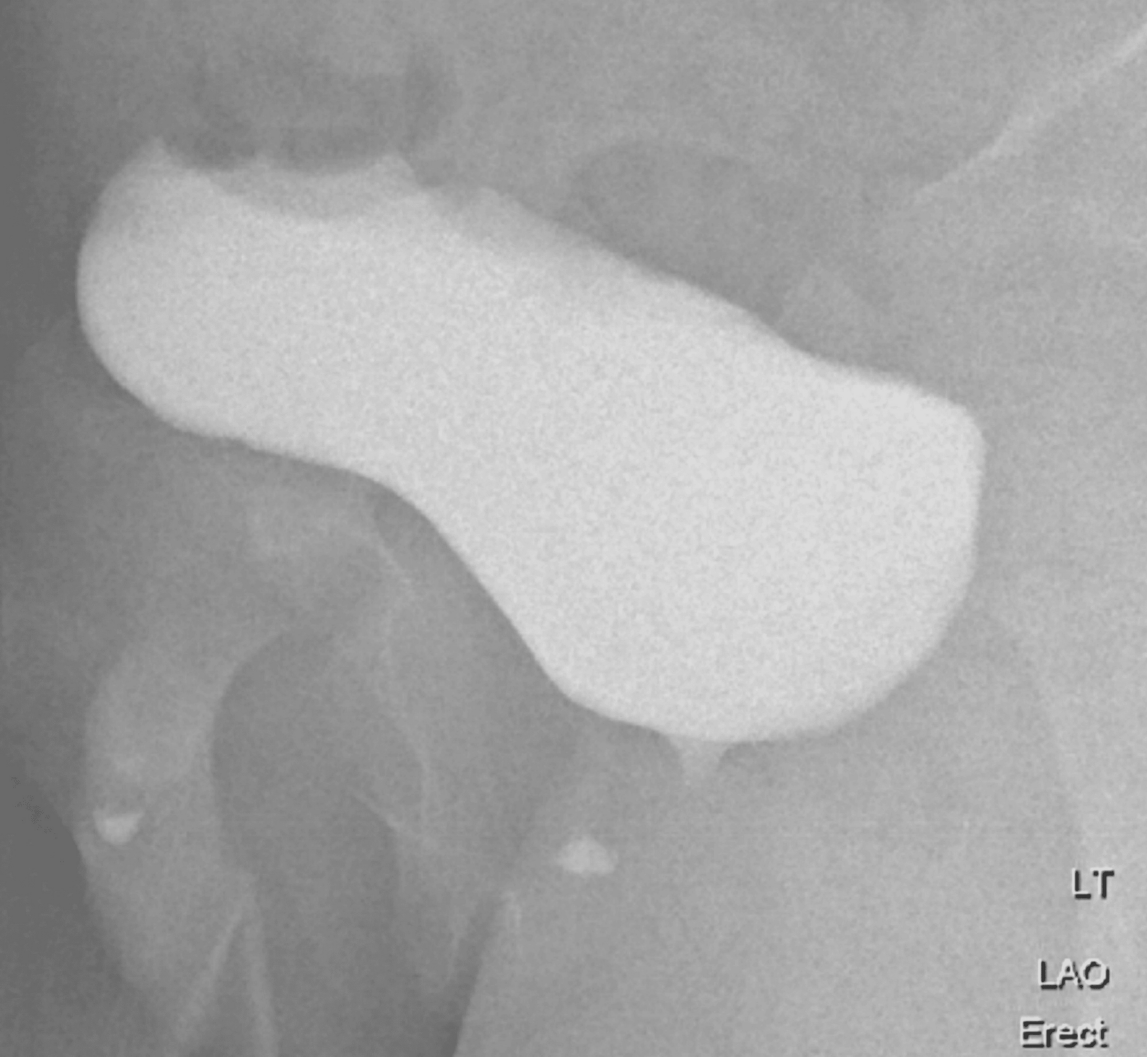
Micturating cystourethrogram showed a large amount of residual urine in the urinary bladder after micturition.

The mass centered at the body and right ala of the S1 vertebra showed involvement of the S1 posterior element. Complete obliteration of the dural sac with resultant compression of traversing cauda equina nerve roots noted (red arrow). Anteriorly, there is also a sizable extraosseous pelvic soft tissue component (blue arrow). Moreover, the tumor signal showed extension along the right L5/S1 intervertebral foramen, bilateral S1, and right S2 sacral neuroforamina (Figure 3).

**Figure 4:**
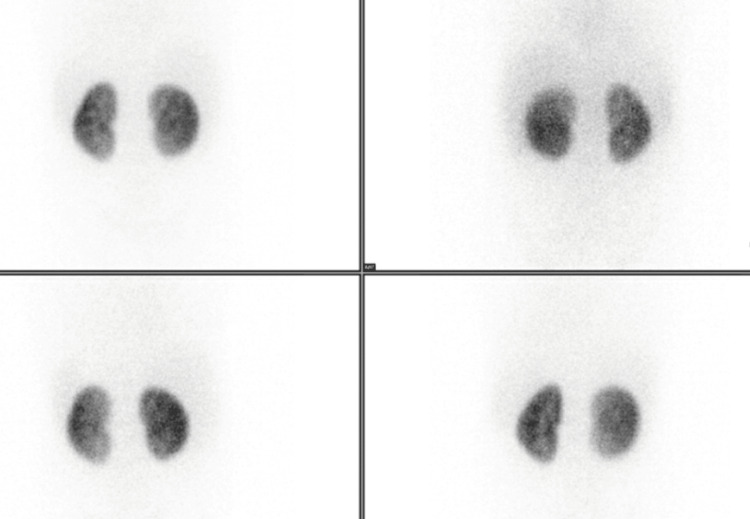
Dimercaptosuccinic acid (DMSA) scan demonstrates symmetric and homogeneous tracer uptake by bilateral kidneys. Normal differential function with no evidence of renal cortical scarring. Differential renal cortical mass ratio, left:right = 48%:52%

A normal micturating cystourethrogram should have no residual post-void urine (Figure 4).

A differential function of 45-55% per kidney is considered normal. Renal cortical scarring will appear as areas of decreased cortical uptake of radiotracer. When such uptake defects are identified, additional imaging modalities, such as ultrasonography or computed tomography, may be used for further evaluation.

Clinical diagnosis of cauda equina syndrome was made. Emergency surgical decompression of the dural sac with laminectomy, tumor biopsy, and debulking were done. Histopathology showed Ewing sarcoma, with EWSR1-FLI1 chimeric transcript detected.

**Figure 5:**
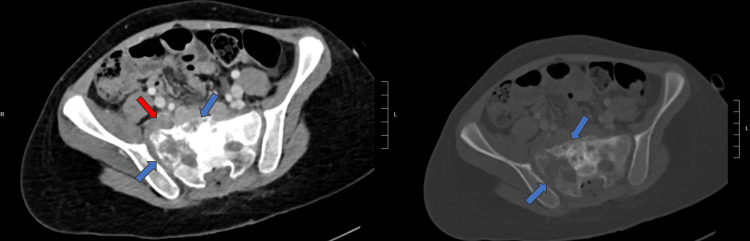
CT thorax, abdomen, and pelvis showing postoperative changes. Index sacral lesion appears predominantly lytic (blue arrow) with involvement of the right sacral neuroforamina and probably right sacroiliac joint. Pelvic extraosseous soft tissue also noted (red arrow).

Postoperative CT thorax, abdomen, and pelvis shows no distant metastasis (Figure 5). Pelvic tumors show a much higher rate of distant spread (25-30%) than extremity tumors (<10%) [6].

The patient was started on Ewing sarcoma treatment as per the Euro Ewing 2012 protocol [7]** **that included systemic chemotherapy and proton radiotherapy as local treatment.

**Figure 6:**
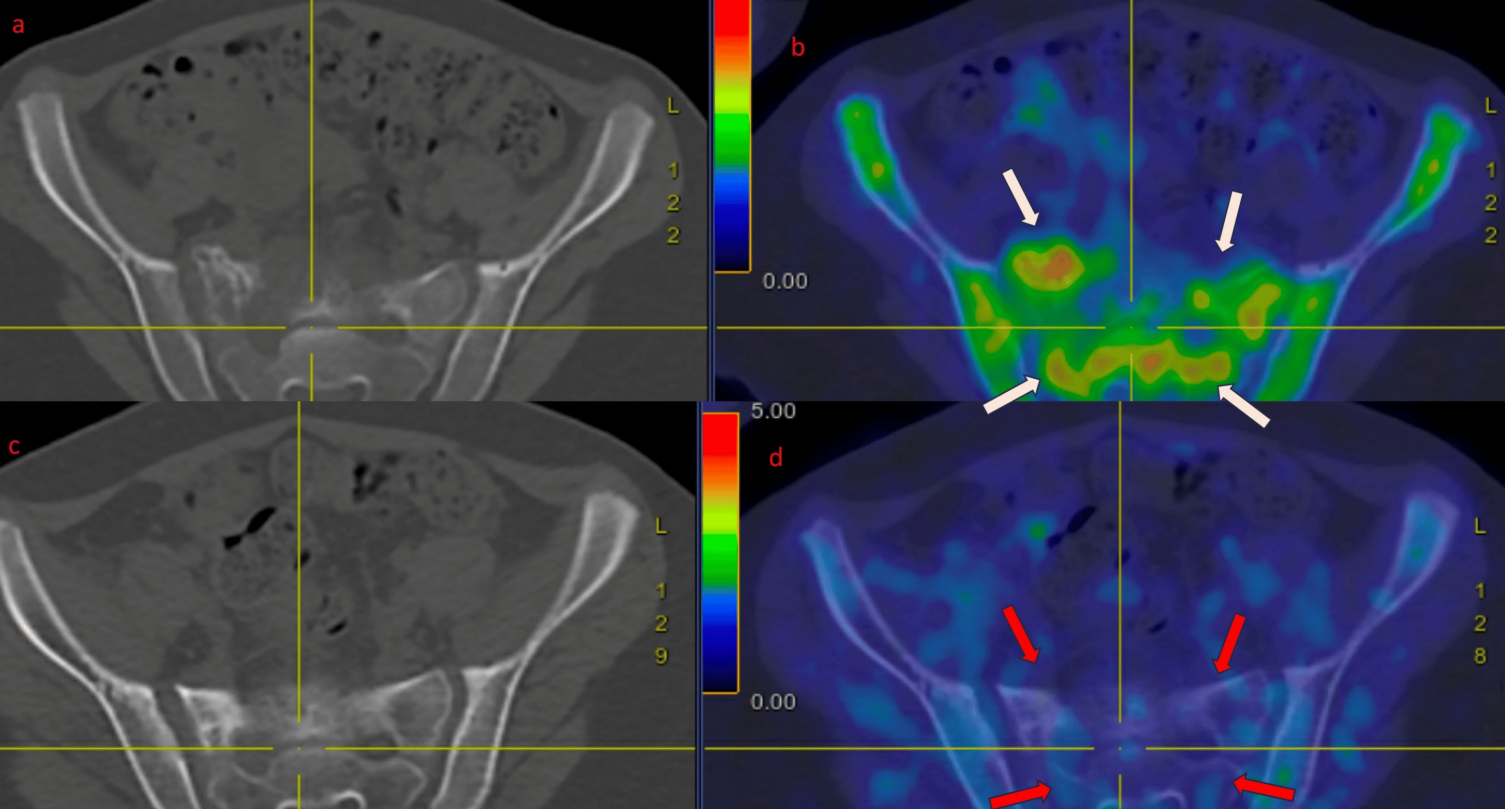
Two PET-CTs performed before starting treatment (a, b) and six weeks after completion of treatment (c, d). Increased uptake corresponding to osseous and extraosseous lesions in pre-treatment PET-CT (white arrows). No avidity was seen in post-treatment PET-CT (red arrows).

Pre- and post-treatment PET-CT scans were performed in the same center (Figure 6). Post-treatment follow-up PET-CT shows residual osseous sclerotic lytic changes with no increased avidity. No new hypermetabolic nodal or distant lesion was noted.

**Figure 7:**
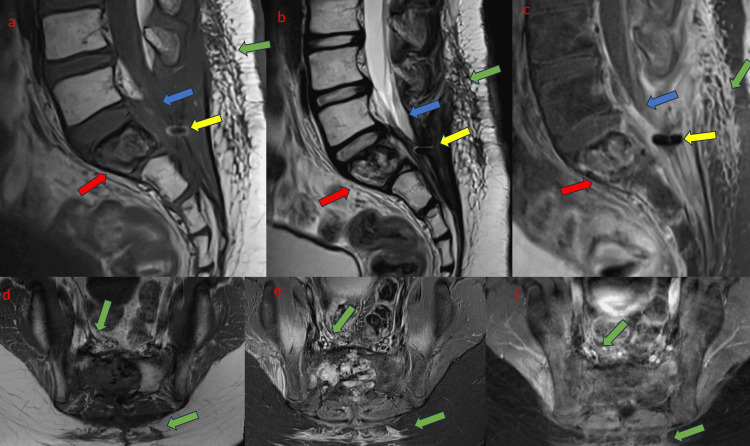
Selected sagittal (a-c) and axial (d-f) MRI images (left to right: T1W, T2W, and T1W post contrast) done 3.5 months after the completion of treatment. Heterogenous sacral lesion shows interval reduction in size. No narrowing of the spinal canal or compression of the cauda equina (blue arrow). Post-treatment changes noted in the pelvis, spinal canal, and posterior sacral region (green arrow). No more enhancing extraosseous soft tissue component noted at the anterior aspect of S1 (red arrow). Metallic artefact related with surgical decompression (yellow arrow).

MRI showed post-treatment changes. However, there was no longer spinal canal narrowing/compression of the cauda equina (Figure 7). The extraosseous soft tissue component in the pelvis was also not seen.

**Figure 8:**
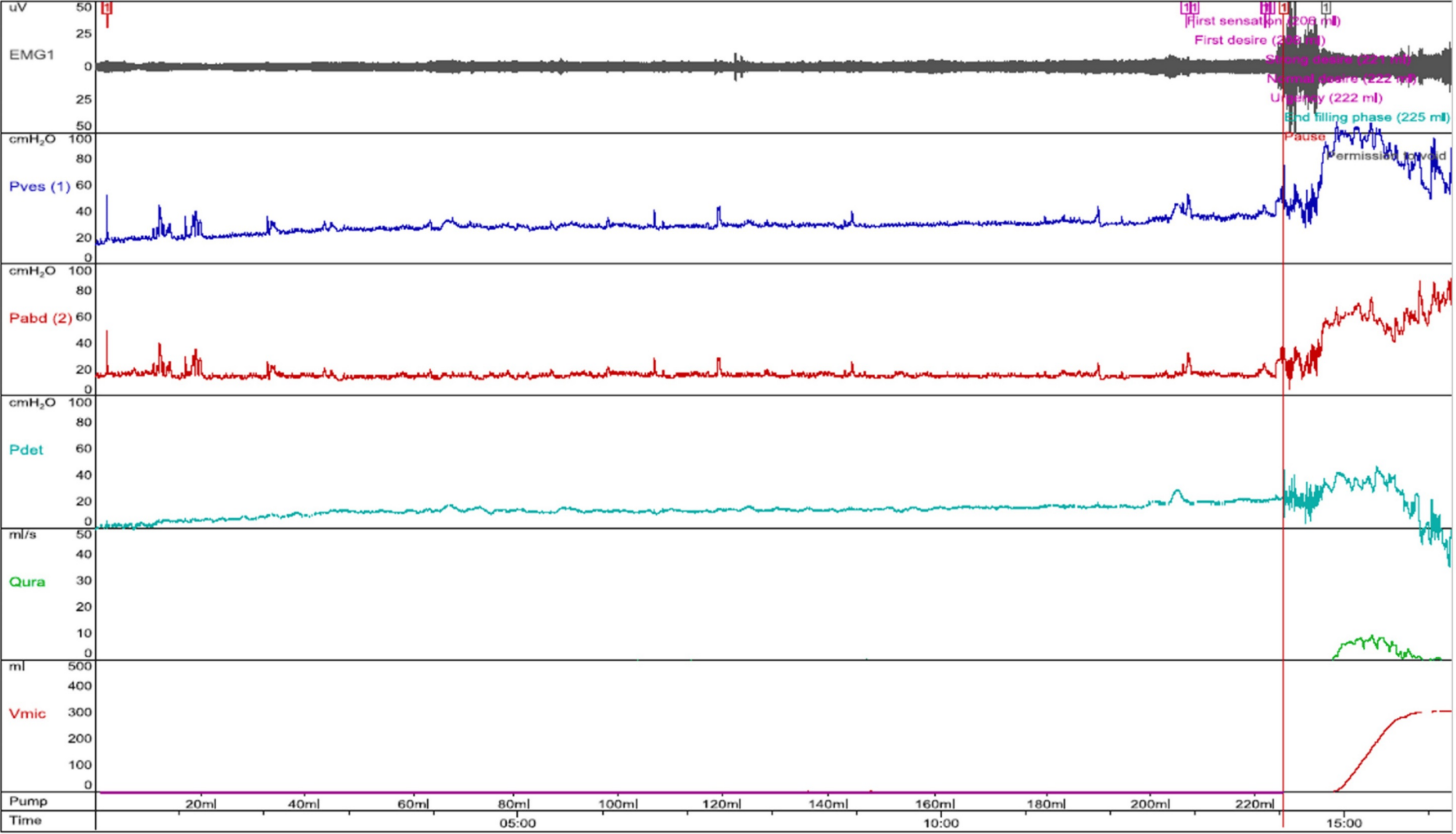
Urodynamic study showing slightly increased bladder capacity. Compliance fair, no significant detrusor overactivity, and fair bladder contraction with satisfactory emptying.

Clinically, the patient demonstrated significant improvement (Figure 8). Although clean intermittent catheterization was required immediately after surgery, the patient later regained the ability to void spontaneously. Bowel management was maintained with regular Fleet enemas. Neurological function returned to normal, with no residual symptoms and normal muscle tone and lower limb strength. The patient will continue to undergo surveillance imaging (PET‑CT and MRI) along with clinical follow‑up to assess for remission.

## Discussion

This case demonstrates sacral Ewing sarcoma presenting with cauda equina syndrome and highlights how multiple imaging modalities contribute to timely diagnosis, treatment planning, and ultimately prognosis. Radiography, although often used as an initial screening tool, tends to underestimate or completely miss the underlying pathology.

MRI is the gold standard for confirming clinically suspected cauda equina syndrome. In this case, significant spinal canal narrowing and compression of cauda equina secondary to sacral Ewing sarcoma accounted for clinical suspicion of cauda equina syndrome. The presence or absence of bowel and bladder symptoms distinguishes incomplete cauda equina syndrome from the complete form [8-10]. Cauda equina syndrome is a neurological emergency that requires surgical decompression, debulking in this case. The most favorable results appear to occur when surgical decompression is performed within 24 hours [8]. Outcomes tend to be less favorable for individuals with complete cauda equina syndrome [11]. In this case, after treatment, with a decrease in tumor bulk, there was resolution of cauda equina compression and subsequently cauda equina syndrome.

MRI is done to evaluate the local extent of the tumor. CT (horax, abdomen, and pelvis) or PET-CT is done to look for distant metastasis and also the local extent of the tumor. Pelvic tumors show a much higher rate of distant spread (25-30%) than extremity tumors (<10%), with metastases occurring about equally often to the lungs and bones, and the spine being the most frequent site of bone involvement [1,6,12]. The presence of distant metastases at the time of diagnosis strongly influences prognosis, worse in metastatic disease. Interval MRI +/- PET-CT is used post treatment to assess for treatment response, surveillance, and remission assessment.

Micturating cystourethrogram and urodynamic study provide anatomic and functional imaging during filling and micturition. A large amount of residual urine in the urinary bladder after micturition is seen in a neurogenic bladder. Neurogenic bladder can lead to high intravesical pressure (detrusor-sphincter dyssynergia or poor compliance), vesicoureteral reflux, and recurrent urinary tract infections, which are responsible for renal scarring. Scarring can progress to chronic kidney disease and hypertension. The primary purpose of the DMSA scan is to detect and monitor renal scarring, quantify differential renal function, and guide the intensity of bladder management.

Treating sacral Ewing sarcoma is highly complex due to the tumor's location near critical nerves and structures. The standard of care is a multidisciplinary approach that combines systemic chemotherapy with local control, typically either surgery, radiation, or both. In this case, the patient was started on Ewing sarcoma treatment as per the Euro Ewing 2012 protocol [7]. Proton radiotherapy is a highly precise and increasingly preferred treatment for sacral Ewing sarcoma. It allows for higher and more effective doses to be delivered directly to the sarcoma and significantly reduces radiation exposure to the surrounding organs at risk [13,14].

## Conclusions

This relatively rare case of sacral Ewing's sarcoma presenting with cauda equina syndrome illustrates the diagnostic and therapeutic challenges encountered in the pediatric population. Cauda equina syndrome constitutes a neurological emergency, necessitating prompt imaging and urgent surgical decompression. Despite the initially extensive local appearance of the tumor, a favorable prognosis was achieved with appropriate multimodal therapy, including surgery, chemotherapy, and proton radiotherapy. This case further underscores the critical role of imaging, which is indispensable not only for diagnosis but also for planning treatment, monitoring response to therapy, and conducting follow-up surveillance.
